# Na_V_1.5 or K_Ca_2 channel blockade does not increase arrhythmia risk in hypokalemic rabbit hearts, unlike K_V_11.1 inhibition with dofetilide

**DOI:** 10.1016/j.ijcha.2025.101699

**Published:** 2025-05-07

**Authors:** Yannan Yan, Lea Abildgaard, Mark Alexander Skarsfeldt, Sofia Hammami Bomholtz, Ulrik Sørensen, Anders Gaarsdal Holst, Morten Grunnet, Jonas Goldin Diness, Bo Hjorth Bentzen

**Affiliations:** a Department of Biomedical Sciences, University of Copenhagen, Denmark; b Acesion Pharma, Denmark

**Keywords:** KCa2 channels, SK channels, Hypokalemia, QT-interval, Pharmacology, Arrhythmia

## Abstract

**Aims:**

The small conductance calcium activated potassium channel (*KCNN1-3*; K_Ca_2.1–3) is recognized as a possible new anti-arrhythmic drug target for treatment of atrial fibrillation (AF). The aim of this study is to investigate potential ventricular effects of K_Ca_2 channel inhibition under normal, bradycardic and hypokalemic conditions and compare these to classical class I and III anti-arrhythmic drugs.

**Methods and results:**

Rabbit hearts were isolated, AV-ablated, mounted in an ex vivo Langendorff preparation and perfused with normokalemic (4 mM K^+^) Krebs-Henseleit solution, followed by perfusion with drug (AP14145 3 µM; AP30663 1.5 µM; dofetilide 10 nM; flecainide 1.5 µM) or vehicle control. The perfusion was then changed to hypokalemic solution (2.5 mM K^+^) in presence of drug. Changes in ventricular action potential duration were assessed by monophasic action potential recordings. Neither of the K_Ca_2 channel inhibitors (AP14145 or AP30663) or flecainide (Na_V_1.5 inhibitor) prolonged ventricular action potential duration (APD90) or increased pro-arrhythmic markers, whereas dofetilide (K_V_11.1 blocker) prolonged APD and increased the susceptibility to ventricular arrhythmia.

**Conclusions:**

These findings suggests that K_Ca_2 channels have minimal importance for ventricular repolarization in healthy rabbit hearts under both normo- and hypokalemic conditions.

## 1 Introduction

The small conductance calcium activated potassium channel (SK1-3, K_Ca_2.1–3) is recognized as a possible new anti-arrhythmic drug target for treatment of atrial fibrillation (AF) [1]. Recently a phase II clinical trial demonstrated AF cardioversion efficacy of the K_Ca_2 channel inhibitor AP30663 in patients with recent-onset AF episodes [2]. In the heart, K_Ca_2 channels appear to have a larger functional role in the atria as compared to ventricles [[3], [4], [5], [6], [7], [8], [9], [10], [11]]**.** However, preclinical studies have found that this might change under pathophysiological conditions such as heart failure, myocardial infarction or hypokalemia [[12], [13], [14], [15], [16], [17], [18]]. Hypokalemia (Serum K^+^< 3.5 mM) is commonly encountered in the clinic [19], and is a known risk factor for developing the ventricular polymorphic tachycardia called torsade de pointes. Hypokalemia reduces Na^+^/K^+^-ATPase activity and suppresses repolarizing cardiac K^+^ currents. The weakened repolarization reserve and prolonged cardiac action potential promote early after-depolarizations and ventricular arrhythmia. Hypokalemia and long action potentials also increase intracellular Ca^2+^ [20]. Elevated intracellular Ca^2+^ may activate ventricular K_Ca_2 channels, potentially offering protection against arrhythmias during hypokalemia. Another risk factor for triggering arrhythmia is bradycardia, especially in the setting of class III anti-arrhythmic drugs, which can cause reverse use-dependent prolongation of the ventricular action potential duration [21].

The aim of this study is to investigate potential ventricular effects of K_Ca_2 channel inhibition under normal, bradycardic and hypokalemic conditions and compare this to classical class I and III anti-arrhythmic drugs.

## 2 Material and methods

### 2.1 The Langendorff perfused heart procedure

The Langendorff perfused heart experiments were performed under license from the Danish Ministry of justice (2017-15-0201-01296) in accordance with the Danish guidelines for animal experiments according to the European Commission Directive 2010/63/EU. All animals were housed in cages with free access to water and food at the animal house facility at room temperature (21 ◦C) and subjected to a 12-hour light/dark cycle.

A total of 42 female New Zealand White rabbits (1600–2000 g, Charles River, Saint-Germain-Nuelle, France) were used. 11 were excluded from the study because of problems with the operation, failure to maintain adequate perfusion pressure, ischemia during stabilization, and failure to achieve an adequate AV block. Rabbits were anesthetized with an initial i.m. injection of Ketamin 35 mg/kg + xylazin 10 mg/kg. After sedation, heparin (0.5 mL/kg Heparin 1000 IU/mL) was injected in the marginal ear vein and the rabbit was euthanized by i.v. injection of pentobarbital/lidocaine (200 mg/kg)/20 mg/kg. Following euthanasia, each heart was rapidly excised and transferred to a beaker containing chilled Krebs-Henseleit buffer. The heart was cannulated via the aorta in a petri dish containing chilled Krebs-Henseleit buffer (in mM: NaCl 120, NaHCO_3_ 25, KCl 4, MgSO_4_ 0.6, NaH_2_PO_4_ 0.6, CaCl_2_ 2.5, glucose 11) and connected to the Langendorff setup (Hugo Sachs Elektronik, Harvard Apparatus GmbH, March, Germany). The heart was then retrogradely perfused at a constant perfusion pressure of 80 mmHg at 37 °C, pH 7.4 with Krebs-Henseleit buffer saturated with 95 % O_2_ and 5 % CO_2_. For hypokalemic perfusion the K^+^ level was adjusted to 2.5 mM.

The heart was immersed in a temperature-controlled bath containing Krebs-Henseleit buffer. The heart was mechanically AV ablated by scarring the area known as the “triangle of Koch” of the right atrium using surgical scissors and left to stabilize until the ventricular rate was < 90 BPM. The hearts were excluded if the ventricular rate at the end of stabilization dropped below 30 BPM. An epicardial monophasic action potential electrodes (MAPs) was positioned on the left ventricle to record left ventricular action potential durations (APD_90_). A bipolar pacing electrode was placed on the left ventricle for epicardial ventricular pacing. The hearts were stimulated using square pulses of 2 ms durations at five times diastolic threshold for excitation at basic cycle lengths of 650, 500 and 300 ms (92 BPM, 120 BPM and 200 BPM). Testing for electrically induced arrhythmias was done by giving 10 stimuli (S1) at a given pacing rate and then applying an extra-stimulus (S2). The S1-S2 interval was increased until the S2 induced an action potential. One to two action potentials were induced by S2 stimulation at each paced heart rate and in some cases, this gave rise to a VF episode.

Signals were sampled at a frequency of 2 k/s and converted by a 16/30 data acquisition system from PowerLab systems (ADInstruments, Oxford, UK) and monitored using LabChart 7 software (ADInstruments).

### 2.2 Study Design

**Fig. 1:**
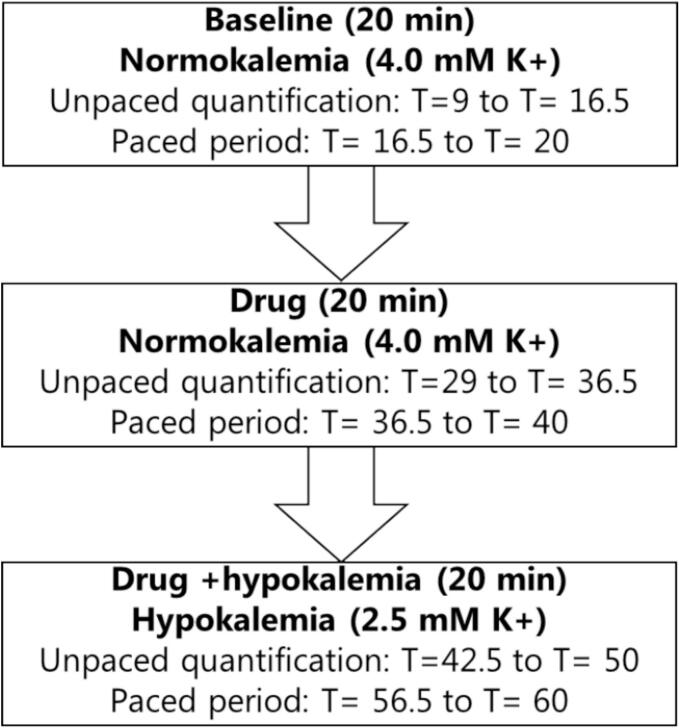
**Timeline for experiment**. Note that the arrhythmia quantification period during hypokalemia is placed 2.5 min after changing the solution to hypokalemic conditions in contrast to drug treatment where unpaced quantification was performed after 9 min. This is done to assess the immediate effect of hypokalemia.

Before the experiment start, each heart was randomized to one of 5 treatment groups: time matched control treated with DMSO (TMC, n = 8), AP14145 (n = 6), AP30663 (n = 6), dofetilide (n = 6) or flecainide (n = 5, one heart excluded post-experiment because of bad MAP signals). After stabilization (15–25 min), the experiment consisted of three subsequent 20 min periods (Fig. 1): 1) baseline recording in normokalemia (4 mM K^+^), 2) drug (AP14145, AP30663, flecainide, dofetilide or DMSO), and 3) drug + hypokalemia (2.5 mM K^+^). Each period consisted of an unpaced period that was used to assess spontaneous arrhythmia and beat-to-beat variability of the APD_90_, and a paced period where APD, APD triangulation (APD_90_-APD_30_), VERP, and electrical induction of arrhythmia were assessed (see Fig. 1).

### 2.3 Arrhythmia scoring

The ventricular action potential duration (APD) recordings from the three perfusion periods were analyzed for incidences of early and delayed afterdepolarizations (EADs and DADs, respectively) and ventricular tachycardia/ventricular fibrillation.

A predefined arrhythmia scoring system was used to score the severity of the arrhythmia see Table 1. The arrhythmia score is the sum of the individual scores.

**Table 1:** **Arrhythmia scoring system**. The arrhythmia score can be in the range from 0 to 32 (5 + 5+(12 + 10)). * If both electrically and spontaneous VF occurred a score of 10 was used.

Arrhythmia	Score
Number of EADs(0.1 point/EAD)	e.g. 35 EADs will give a score of 3.5 (max 5)
Number of DADs (0.1 point/DAD)	e.g. 7 DADs will give a score of 0.7 (max 5)
VF duration (s) (0.1 point/s VF)	e.g. 40 s will give a score of 4 (max 12)
S1-S2 induced VF	5
Spontanous VF	10*

### 2.4 Data analysis

All raw data analysis was performed in a blinded fashion by two operators, where the operators were blinded to the treatment group. The mean of the two operators’ findings were used for further statistical analysis. Data and figures were analyzed using Prism 9 (GraphPad Software, CA, USA). Beat-to-beat variability of APD_90_ was calculated at a period of maximal heart rate during the unpaced quantification period (see Fig. 1). The short-term variability (STV) was calculated using the formula: STV = $\sum \frac{\left|D_{n+1}-D_{n}\right|}{X\times \surd 2}$ where D_n_ is the APD_90_ of beat n and X is the number of beats. Triangulation was calculated as the average difference in duration between APD_90_ and APD_30_ of 10–50 consecutive paced beats.

Changes from baseline value (Δ-value) for APD90, triangulation, STV and arrhythmia score was calculated for each group (TMC, AP14145, AP30663, dofetilide, flecainide) at each perfusion period (drug and drug + hypokalemia). Using a two-way ANOVA or, in case of missing values, a mixed-effects analysis with Sidak’s multiple comparisons post hoc test a comparison of Δ-values between TMC and treatment groups was performed. P values are given with three decimals.

### 2.5 Drugs and chemicals

Unless otherwise mentioned, all the chemicals used were of analytical grade and were obtained from Sigma-Aldrich. AP14145 (IC_50_ on SK3 = 1.1 µM [22]) and AP30663 (IC_50_ on SK3 = 1.1 µM [11]) were synthesized at Syngene, India, and dissolved in DMSO as concentrated stock solutions of 10  mM. Flecainide and dofetilide were dissolved in DMSO as concentrated stock solution of 10 mM and 100 µM respectively. The stock solutions were stored at −21 °C until used.

30 μM AP14145 and 1.5 µM AP30663 were used for the study. The chosen concentrations correspond to the anticipated maximal free plasma concentration in in vivo and human studies respectively. Dofetilide was used in 10 nM, which represents the high end of a calculated free plasma concentration when treated twice daily with 500 mg. Steady state plasma concentrations of dofetilide when treated with 500 mg twice daily is from 2-7 ng/mL. Assuming a molecular weight of 441 mg/mmol and a plasma binding of 60 %, this corresponds to a calculated free plasma concentration of 6 nM (https://www.accessdata.fda.gov/drugsatfda_docs/label/2016/020931s012s013lbl.pdf). 1.5 µM flecainide was used. According to Boehringer-Ingelheim‘s drug information patients successfully treated with flecainide had a plasma level between 0.2 and 1 µg/mL (40 % plasma bound), which correspond to a free plasma concentration of 1.5 µM

## 3 Results

### 3.1 K_Ca_2 channel inhibitors do not prolong ventricular APD_90_ in rabbits

**Fig. 2:**
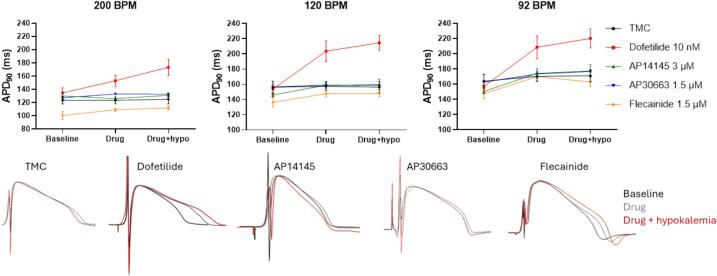
**Effects on ventricular APD_90_.** Top: The effects of DMSO (black), dofetilide (red), AP14145 (green), AP30663 (blue), and flecainide (orange) on ventricular APD_90_ measured at 3 different heart rates (200, 120 and 92 BPM), during normo- and hypokalemia. Data are presented as mean ± SEM, n = 5–8, details on sample sizes see Table 2. Bottom: Mono phasic action potential recordings from the 5 different groups (TMC, dofetilide, AP14145, AP30663 and flecainide) during baseline (black), drug (grey) and drug + hypokalemia (red) paced at 92 BPM. (For interpretation of the references to colour in this figure legend, the reader is referred to the web version of this article.)

Action potential duration (APD) varies with heart rate [23]. As expected, we found that decreasing the heart rate prolongs the ventricular action potential duration (see Fig. 2 ABC at baseline; 128 ± 3 ms vs. 159 ± 4 ms at 200 BPM and 92 BPM respectively, p < 0.001). K_V_11.1 inhibition by dofetilide significantly prolonged the APD_90_ when measured at 92 and 120 BPM under normokalemic conditions and at all heart rates during hypokalemic conditions (Table 2, Fig. 2). Like other K_V_11.1 blockers, dofetilide is known to demonstrate reverse use-dependence (i.e. relatively larger APD prolongations at slower heart rates). We also observed this (Table 2, Fig. 2). While hypokalemia alone did not affect APD_90_, the effect of dofetilide on APD_90_ was exacerbated by hypokalemia at all pacing rates. No significant effects on ventricular repolarization were found for any of the two K_Ca_2 channel inhibitors AP14145 and AP30663 or flecainide (see Table 2, Fig. 2) when compared to time matched controls (TMC) under both normo- and hypokalemic conditions.

**Table 2:** **Changes from baseline in APD_90_ for each group, measured at different pacing rates.** P-values refer to the comparison of Δ-values between TMC and treatment groups. For sample size see column (n). The values correspond to the number of animals with analyzable recordings from 200 BPM, 120 BPM and 92 BPM.

	**Δ-APD_90_ (ms)** **Normokalemia**			**n**	**Δ-APD_90_ (ms)** **Hypokalemia**			**n**
	***200 BPM***	***120 BPM***	***92 BPM***		***200 BPM***	***120 BPM***	***92 BPM***	
**TMC**	0 ± 6	1 ± 6	6 ± 8	8	2 ± 7	0 ± 7	7 ± 7	8
**Dofetilide** **10 nM**	18 ± 6 (p = 0.175)	50 ± 10 (p < 0.001)	51 ± 10 (p < 0.001)	6	40 ± 17 (p = 0.004)	56 ± 9 (p < 0.001)	60 ± 12 (p < 0.001)	5
**AP14145** **3 µM**	−4 ± 2 (p = 0.980)	13 ± 3 (p = 0.756)	24 ± 5 (p = 0.307)	6	1 ± 5 (p = 0.999)	14 ± 5 (p = 0.533)	27 ± 7 (p = 0.206)	6
**AP30663** **1.5 µM**	6 ± 7 (p = 0.935)	3 ± 4 (p = 0.999)	9 ± 8 (p = 0.997)	6, 6, 5	6 ± 7 (p = 0.977)	2 ± 2 (p = 0.997)	12 ± 7 (p = 0.997)	6, 6, 5
**Flecainide** **1.5 µM**	8 ± 6 (p = 0.817)	11 ± 5 (p = 0.862)	22 ± 2 (p = 0.435)	5	11 ± 7 (p = 0.749)	11 ± 8 (p = 0.815)	16 ± 8 (p = 0.883)	5

### 3.2 Dofetilide but not K_Ca_2 inhibitors or flecainide increases pro-arrhythmic markers

**Fig. 3:**
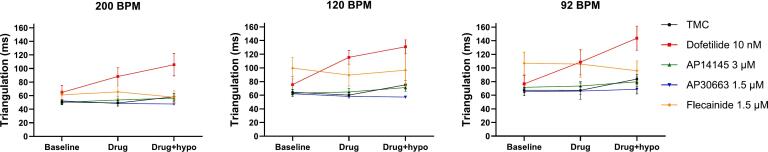
**Effects on ventricular action potential triangulation.** The effects of DMSO (black), dofetilide (red), AP14145 (green), AP30663 (blue), and flecainide (orange) on ventricular action potential triangulation (APD30-PAD90) measured at 3 different heart rates (200, 120 and 92 BPM), during normo- and hypokalemia. Data are presented as mean ± SEM, n = 5–8, details on sample sizes see Table 4. (For interpretation of the references to colour in this figure legend, the reader is referred to the web version of this article.)

Hypokalemia has been found to increase the risk of ventricular arrhythmia. Triangular ventricular action potentials and increased beat-to-beat variability of APD are pro-arrhythmic markers. In accordance, we observed that in the TMC group hypokalemia was associated with significantly more triangular APD as compared to the APD shape recorded in normokalemia, especially at lower heart rates (Table 3 and Fig. 3).

**Table 3:** **Effects of hypokalemia on ventricular action potential triangulation (APD30-APD90) at different heart rates.** P-values refer to paired Student’s t-tests between TMC hearts at normo- and hypokalemia at different heart rates**.**

	**Triangulation (ms)**		**Average** **difference (ms)**	**P value**
**Pacing rate**	***DMSO***	***Hypokalemia***		
**200 BPM**	49 ± 5	58 ± 5	9 ± 3	0.017
**120 BPM**	60 ± 4	75 ± 6	15 ± 4	0.005
**92 BPM**	67 ± 6	84 ± 7	16 ± 4	0.005

**Fig. 4:**
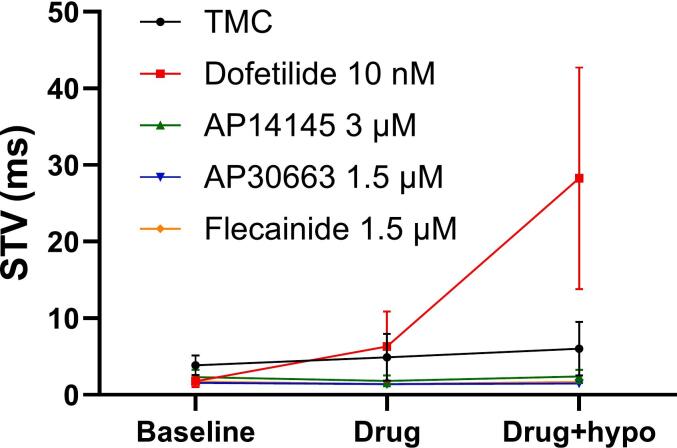
**Effects of drugs on short-term variability of ventricular APD90**. The effects of DMSO (black), dofetilide (red), AP14145 (green), AP30663 (blue), and flecainide (orange) on short-term variability (STV) of ventricular APD90 Data are presented as mean ± SEM, n = 5–8, details on sample sizes see Table 5. (For interpretation of the references to colour in this figure legend, the reader is referred to the web version of this article.)

Similarly, perfusion with dofetilide caused a significant triangulation of the APD as compared to the effects observed in the TMC group. This effect of dofetilide was exacerbated by hypokalemia and was more pronounced at slower heart rates (Table 4 and Fig. 3). Dofetilide alone did not significantly affect beat-to-beat variability, but in combination with hypokalemia the beat-to-beat variability dramatically increased as compared to TMC (Fig. 4 and Table 5). K_Ca_2 channel inhibitors and flecainide neither affected APD triangulation nor beat-to-beat variability addressed as short-term variability (STV) under normokalemic or hypokalemic conditions.

**Table 4:** **Change from baseline in APD triangulation for each group, measured at different pacing rates.** P-values refer to the comparison of Δ-values between TMC and treatment groups. For sample size see column (n). The values correspond to the number of animals with analyzable recordings from 200 BPM, 120 BPM and 92 BPM.

	**Δ-triangulation (ms)** **Normokalemia**			**n**	**Δ-triangulation (ms)** **Hypokalemia**			**n**
	***200 BPM***	***120 BPM***	***92 BPM***		***200 BPM***	***120 BPM***	***92 BPM***	
**TMC**	−2 ± 4	−4 ± 4	0 ± 5	8	7 ± 4	11 ± 6	16 ± 5	8
**Dofetilide** **10 nM**	25 ± 6 (p = 0.007	40 ± 8 (p < 0.001)	32 ± 16 (p = 0.026)	5, 6, 6	45 ± 16 (p < 0.001)	60 ± 9 (p < 0.001)	73 ± 8 (p = 0.006)	5, 5, 5
**AP14145** **3 µM**	3 ± 3 (p = 0.927)	2 ± 3 (p = 0.957)	2 ± 5 (p = 0.999)	6	6 ± 6 (p = 0.997)	8 ± 6 (p = 0.998)	8 ± 5 (p = 0.901)	6
**AP30663** **1.5 µM**	−4 ± 3 (p = 0.997)	−4 ± 5 (p > 0.999)	−1 ± 9 (p = 0.999)	6, 6, 5	−6 ± 6 (p = 0.237)	−5 ± 4 (p = 0.421)	−1 ± 3 (p = 0.453)	6, 6, 5
**Flecainide** **1.5 µM**	4 ± 6 (p = 0.891)	−10 ± 10 (p = 0.961)	−1 ± 8 (p = 0.999)	5	−4 ± 6 (p = 0.493)	−3 ± 21 (p = 0.581)	−11 ± 12 (p = 0.096)	5

**Table 5:** **Change from baseline of short-term variability of ventricular APD.** P-values refer to the comparison of Δ-values between TMC and treatment groups. For sample size see column (n). The values correspond to the number of animals with analyzable recordings.

	**Δ-STV (ms)** **Normokalemia**	**n**	**Δ-STV (ms)** **Hypokalemia**	**n**
**TMC**	1 ± 2	8	3 ± 4	7
**Dofetilide 10 nM**	6 ± 6 (p = 0.999)	5	27 ± 15 (p = 0.005)	5
**AP14145 3 µM**	0 ± 1 (p = 0.998)	6	0 ± 2 (p = 0.904)	5
**AP30663 1.5 µM**	0 ± 1 (p = 0.999)	6	0 ± 1 (p = 0.896)	6
**Flecainide 1.5 µM**	0 ± 1 (p = 0.999)	5	0 ± 1 (p = 0.914)	5

### 3.3 Dofetilide, but not K_Ca_2 channel inhibitors or flecainide results in more ventricular arrhythmia

**Fig. 5:**
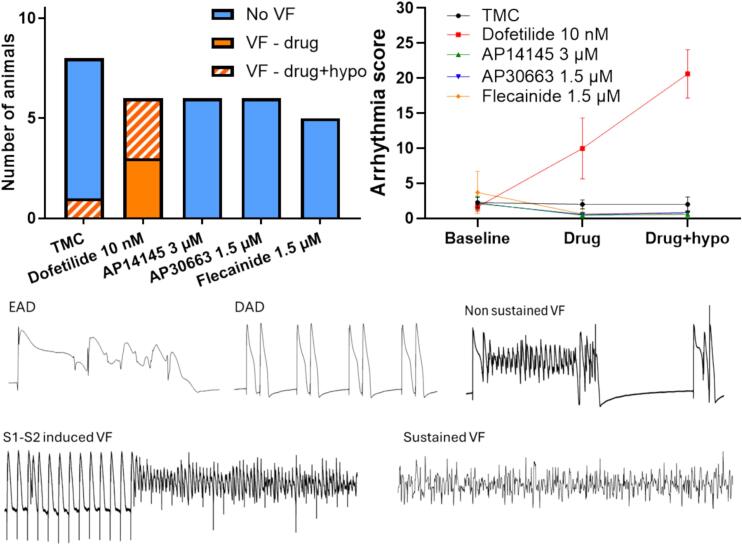
**Effect of drugs on ventricular arrhythmia.** Left: Number of animals with one or more VF episodes during normo- or hypokalemia. Right: Arrhythmia scores for the different treatment groups during normo- or hypokalemia (data are presented as mean ± SEM). Bottom: Examples of different arrhythmia.

Perfusion with dofetilide alone and in combination with hypokalemia provoked VF episodes in all animals, with one heart going into sustained VF. In comparison only 1 out 8 hearts developed non-sustained VF in the TMC group, and no VF (sustained or non-sustained) occurred or could be provoked in the hearts treated with K_Ca_2 inhibitors or flecainide (Fig. 5 left). The pro-arrhythmic effect was also observed on the arrhythmia score (Fig. 5 right). Here dofetilide alone and in combination with hypokalemia resulted in significantly higher arrhythmia scores than in the TMC group. Although one episode of VF was observed in the TMC group during hypokalemia, hypokalemia *per se* did not affect the overall arrhythmia score (Fig. 5 and Table 6). The arrhythmia score for the K_Ca_2 channel inhibitor groups and flecainide did not differ significantly from that of the TMC group (Fig. 5 and Table 6).

**Table 6:** **Change from baseline in arrhythmia score for each group.** P-values refer to the comparison of Δ-values between TMC and treatment groups. For sample size see column (n). The values correspond to the number of animals with analyzable recordings.

	**Δ-arrhythmia score** **Normokalemia**	**Δ-arrhythmia score** **Hypokalemia**	**n**
**TMC**	0 ± 1	0 ± 1	8
**Dofetilide 10 nM**	8 ± 5 (p = 0.005)	19 ± 4 (p < 0.001)	6
**AP14145 3 µM**	−2 ± 1 (p = 0.970)	−2 ± 1 (p = 0.963)	6
**AP30663 1.5 µM**	−2 ± 1 (p = 0.980)	−1 ± 1 (p = 0.984)	6
**Flecainide 1.5 µM**	−2 ± 1 (p = 0.752)	0 ± 1 (p = 0.763)	5

## 4 Discussion

The main findings of this study are: 1) Inhibition of the K_V_11.1 channel (hERG) by dofetilide was associated with an increased occurrence of pro-arrhythmic markers and ventricular arrhythmia. This proarrhythmia was exacerbated by hypokalemia. 2) Although K_Ca_2 channels are expressed in rabbit ventricles [15,24], inhibition of these did not result in increased proarrhythmia even when challenged by hypokalemia, similar to what we observed for the sodium channel blocker flecainide.

K_Ca_2 channel inhibitors are in clinical development for treatment of atrial fibrillation [2]. As cardiac safety of anti-arrhythmic drugs is of key importance, we studied how K_Ca_2 channel inhibitors compared to two anti-arrhythmic drugs flecainide (class I, Na_V_1.5 inhibitor) and dofetilide (class III, K_V_11.1 inhibitor) under pro-arrhythmic conditions (bradycardia and hypokalemia), focusing on arrhythmia markers such as triangulation of APD, reverse-use dependence and instability [25].

Hypokalemia is a known risk factor for developing ventricular arrhythmia. Low extracellular K^+^ concentrations reduces the Na^+^/K^+^-ATPase activity, accelerate the inactivation of IKr (K_V_11.1) and delay the recovery from inactivation of Ito (K_V_4.3 + KChIP2) [[26], [27], [28]]. It also stabilizes the blocking cations (Mg^2+^ and polyamines) in the pore of the K_ir_2.1 channel (IK1) [29], and long-term exposure to hypokalemia has also been found to accelerate the internalization and degradation of K_V_11.1 channels [30], collectively decreasing the repolarizing outward K + currents. This prolongs the cardiac action potential, lowers the repolarizing reserve and promotes early-after depolarization generation, which might trigger ventricular tachycardia. In addition, the reduced Na^+^/K^+^-ATPase activity and consequent elevated intracellular Na^+^ concentrations result in lowered Na^+^/Ca^2+^ activity (lowered calcium efflux), which in combination with APD prolongation increases intracellular Ca^2+^ [20,31]. This may also activate CamKII and enhance late-Na_V_^+^ currents [32]. In the setting of hypokalemia further attenuation of the repolarizing reserve by class III antiarrhythmic drugs such as dofetilide increase the risk of arrhythmia [32]. Consequently, dofetilide is contraindicated in people with hypokalemia. We also observed prolonged action potential durations, increased triangulation of APD, instability and propensity to arrhythmia when dofetilide was combined with hypokalemia.

Beat-to-beat variation of cardiac APD is a physiological feature of the heart, but increases in short-term variability of APD is a risk predictor of arrhythmia [33]. The ionic mechanism behind beat-to- beat variability is complex, but stems from multiple parameters including the stochastic gating of cardiac ion channels [34], the finely orchestrated interplay between inward calcium currents and activation of repolarizing K^+^ currents, prolonged APD and abnormal calcium handling [35,36]. It is known that IKr inhibition [35,36] and hypokalemia increase beat-to-beat variability [37]. Interestingly, when combining pharmacological IKr inhibition with hypokalemia we saw a striking synergistic effect on short-term variability. In comparison we saw no changes in short term variability for neither the NaV1.5 inhibitor (flecainide) nor K_Ca_2 channel inhibitors (AP14145 and AP30663).

Because K_Ca_2 channels are activated by increases in intracellular calcium it has been suggested that ventricular K_Ca_2 channels function as a protective mechanism against ventricular arrhythmia during hypokalemia [18,38]. Performing optical mapping of calcium and APD signals on isolated rabbit hearts Chan et al found that hypokalemia elevates intracellular Ca^2+^ levels, and that apamin, a specific K_Ca_2 channel blocker, causes ventricular APD prolongation, discordant APD alternans, steepens the action potential restitution curve and increases susceptibility of pacing-induced ventricular fibrillation. However, as was the case in guinea pigs [39], we did not observe any change in ventricular repolarization upon perfusion of isolated rabbit hearts with K_Ca_2 channel inhibitors under normo- or hypokalemic conditions. Alternans of APD were also not observed, nor were there any effects on beat-to-beat variability, arrhythmia score or VF risk.

The discrepancies between Chan et al. and our study could stem from differences in experimental set-up. Our study used electrical recordings of action potential durations. For optical mapping a mechanical uncoupler is needed to record signals devoid of motion artifacts. The uncoupler, blebbistatin, used by Chan et al., is known to increase ventricular APD, steepen the APD restitution curve and lower the threshold for ventricular fibrillation induction [40]. We speculate that the use of blebbistatin could have impacted the vulnerability of the heart preparations. Moreover, our study investigated small molecule K_Ca_2 channel inhibitors (AP14145 and AP30663) whereas Chan et al. studied the bee venom toxin apamin. Although apamin is a highly selective pore blocker of K_Ca_2 channels, current clinical candidates targeting K_Ca_2 channels are negative allosteric modulators. These modulators act by shifting the calcium sensitivity of the K_Ca_2 channel rather than directly occluding the pore [11]. Given this distinct mechanism of action, it is conceivable that hypokalemia-induced increases in intracellular calcium could differentially influence the ventricular pro-arrhythmic risk associated with negative allosteric modulators compared to pore blockers like apamin.

Another risk factor for triggering ventricular arrhythmia is bradycardia, especially in the setting of class III antiarrhythmic drugs. This is because many class III drugs exhibit reverse use-dependent prolongation of the ventricular APD, thereby excessively prolonging the APD at slow heart rates, which increases the risk of ventricular arrhythmia [21]. Using optical mapping on isolated rabbit hearts, apamin was found to prolong the ventricular APD more at slower heart rates (basic cycle length (BCL) of 1000 ms) [18]. We did not observe any prolongation or triangulation of APD at slow heart rates (BCL of 650 ms) for the tested K_Ca_2 channel inhibitors. Given that fast heart rates increase intracellular calcium and recent studies of increased K_Ca_2 channel activity in the presence of rapid pacing in human atrial cells [41], future investigations of the impact of K_Ca_2 channels for ventricular repolarization during tachypacing would be interesting.

In conclusion in isolated, AV-ablated female rabbit hearts, K_Ca_2 channel inhibition appears to have no proarrhythmic effects under normokalemia as well as when challenged with hypokalemia. These findings suggest that K_Ca_2 channel inhibition by negative allosteric modulators have minimal importance for ventricular repolarization in isolated healthy rabbit hearts under both normo- and hypokalemic conditions.

## 5 Limitations

In the study only female rabbits were used do to constrains of our animal facility. Sex differences in long-QT-related arrhythmias are well documented in the clinical setting and in rabbits [42], and so our findings might not fully apply to male rabbits. AP14145 and AP30663 are small molecule inhibitors of K_Ca_2 channels. Although their ion channel selectivity and off-target profile has been extensively investigated [11,22] off-target effects cannot be excluded, including reported effects on late-Na_V_ albeit at higher concentrations.

Note.

This author takes responsibility for all aspects of the reliability and freedom from bias of the data presented and their discussed interpretation.

Funding

This research did not receive any specific grant from funding agencies in the public, commercial, or not-for-profit sectors.

## CRediT authorship contribution statement

**Yannan Yan:** Writing – original draft, Visualization, Project administration, Methodology, Investigation, Formal analysis, Data curation. **Lea Abildgaard:** Writing – original draft, Visualization, Methodology, Investigation, Formal analysis, Data curation. **Mark Alexander Skarsfeldt:** Writing – original draft, Visualization, Validation, Supervision, Project administration, Methodology, Investigation, Formal analysis, Data curation, Conceptualization. **Sofia Hammami Bomholtz:** Writing – original draft, Validation, Methodology, Investigation, Formal analysis, Data curation. **Ulrik Sørensen:** Resources. **Anders Gaarsdal Holst:** Writing – original draft, Supervision, Resources, Funding acquisition, Conceptualization. **Morten Grunnet:** Writing – original draft, Validation, Supervision, Project administration, Funding acquisition, Data curation, Conceptualization. **Jonas Goldin Diness:** Writing – original draft, Visualization, Validation, Project administration, Methodology, Investigation, Formal analysis, Data curation, Conceptualization. **Bo Hjorth Bentzen:** Writing – review & editing, Writing – original draft, Visualization, Validation, Supervision, Resources, Project administration, Methodology, Investigation, Formal analysis, Data curation, Conceptualization.

## Declaration of competing interest

The authors declare that they have no known competing financial interests or personal relationships that could have appeared to influence the work reported in this paper.
